# The Structure and Properties of Microbial Enzymes

**DOI:** 10.3390/microorganisms12010045

**Published:** 2023-12-27

**Authors:** Stéphane Réty

**Affiliations:** Laboratoire de Biologie et Modelisation de la Cellule, Ecole Normale Superieure de Lyon, CNRS, UMR 5239, Inserm, U1293, Universite Claude Bernard Lyon 1, 46 Allee d’Italie, F-69364 Lyon, France; stephane.rety@ens-lyon.fr

Many microbes are pathogens not only to humans but also to cattle and crops. Some microbial enzymes, which are often different from the host, are of particular interest as targets against pathogens. However, microbes are also a valuable enzyme source, catalyzing specific reactions that can be of economic interest. For instance, microbes have particular enzymes such as the restriction enzyme, polymerase and CRISPR-Cas immune system. The identification of these proteins and rational engineering have allowed the development of molecular biology tools, the applications of which are increasing every day. Because microbes can be more easily selected, engineered and modified by directed evolution, the enzymes they produce are largely used as model enzymes for fundamental research and also in the development of industrial processes. They are also used for environmental remediation, metal bioleaching and water purification [1]. Chemical and thermal robustness are crucial for such applications, and the basis of these properties has been widely deciphered by studying structure–function relationships. New methods for the reliable prediction of structures [2], and also for predicting enzyme kinetic parameters [3], have recently revolutionized the field. In this Special Issue, the practical implementation of these methods for structure and function prediction has led to improvements in the enzymes used for sludge treatment and bioremediation, as well as enzymes used in the industrial process. Structure and function prediction can also help us to understand some fundamental biological pathways, and these findings can be extrapolated to more complex organisms. AI-based algorithms are now widely used in all aspects of prediction and engineering, but there are still some improvements to be achieved, in particular in the prediction of protein–protein and protein–ligand complexes [4]. Establishing a reliable enzymatic mechanism is a difficult task because catalytic reactions have to be precisely described with sub-angstrom precision. Therefore, correct ligand positioning in the active site and protein loops dynamics, usually critical for ligand binding and catalysis, are not trivial steps. Multiscale molecular dynamics, from coarse-grained to QM-MM, ref. [5] can help to improve mechanistic models and compute binding energies and kinetic parameters. Prediction, simulation and computation are crucial in engineering, and their development will accelerate research in microbial enzyme engineering.
